# Superior Mechanical Performance of Inductively Sintered Al/SiC Nanocomposites Processed by Novel Milling Route

**DOI:** 10.1038/s41598-020-67198-w

**Published:** 2020-06-25

**Authors:** Saud M. Almotairy, Nabeel H. Alharthi, Hamad F. Alharbi, Hany S. Abdo

**Affiliations:** 1 Center of Excellence for Research in Engineering Materials (CEREM), King Saud University, P.O. Box 800, Riyadh, 11421 Saudi Arabia; 2 Mechanical Engineering Department, King Saud University, P.O. Box 800, Al-Riyadh, 11421 Saudi Arabia; 3 Mechanical Design and Materials Department, Faculty of Energy Engineering, Aswan University, Aswan, 81521 Egypt

**Keywords:** Synthesis and processing, Composites

## Abstract

This paper explores new routes for flake powder metallurgy, with the aim of designing an effective route for fabricating metal matrix nanocomposites, combining high strength and good ductility. A new route that uses three speeds, instead of the two speeds characterizing the shift-speed ball milling (SSBM) route, has been suggested and implemented. The mechanisms of these routes were illustrated based on the intensity of ball-powder-ball collisions and the morphology evolution. The ball milled powder were characterized using filed emission scanning electron microscope (FESEM), X-ray diffractometer (XRD) and Energy dispersive spectroscopy (EDS) to investigate the morphology evolution of the composites powder and the homogenous distribution of the SiC nanoparticles within the Al matrix. The reinforcing adequacy and interfacial bonding of 2 wt.% SiC nanoparticles in an inductively sintered composite has been investigated. Mechanical testing of the produced bulk composites resulted in achieving superior mechanical properties, characterized by 92% higher hardness, 180% higher yield strength, 101% higher ultimate strength, and 0% loss in uniform elongation, compared with those of regular SSBM. This is attributed to the homogeneous dispersion of the reinforcement into the Al matrix.

## Introduction

There is a great need for advanced materials with superior mechanical and physical properties in several areas, such as in aerospace and military^1,2^. This is attributed to the fact that monolithic materials are generally unable to combine different mechanical properties such as strength and ductility^3^. Metal matrix composites (MMCs) have shown promise in addressing this issue, combining or even altering to the best properties of a strongly reinforced ductile and durable matrix^4–7^. Incorporating reinforcements into the aluminium matrix can enhance the strength, hardness, and both wear and corrosion resistances of the matrix^8,9^. Aluminium matrix composites containing ceramic reinforcements can also exhibit low thermal expansion coefficient, making them as unique materials to be used in defense, cosmonautics, automotive industry, and many other areas^10–12^. SiC is one of the most common particulate reinforcements for strengthening the aluminium matrix^13–15^. However, achieving a significant reduction in the ductility of aluminum MMCs reinforced with SiC microparticles is a key challenge^16–18^. Tjong^19^ stated that, during mechanical loading, the amenability of large ceramic particulates to cracking can lead to low ductility and premature failure. Thus, to overcome these drawbacks, there is a shift toward nanosized reinforcements or the so-called metal matrix nanocomposites (MMNCs) that provide more enhanced mechanical properties^20,21^.

Not only the reinforcement-related factors are affecting the produced MMNCs but also the production routes and their related conditions^22^. Hence, many fabrication routes are utilized under different conditions to synthesis aluminum MMNCs reinforced with nanoparticles. Fabrication routes for manufacturing MMNCs can be classified into two processing routes, namely, solid-state and liquid-state processing. Each of these routes has its own benefits and drawbacks^23^. Liquid-state processing routes, such as stirring, suffer from agglomeration, poor wetting of ceramic nanoparticles with molten metal^24^, and chemical interfacial reaction^22^, limiting their extensive application in fabrication processes. Because of these problems, solid-state manufacturing processes are more favorable^25–27^.

Solid state processes usually include the powder metallurgy (PM) process. In PM, the base metal powder is mixed with reinforcement powder, followed by cold compaction and sintering^5,28^ to form a bulk composite. PM, when utilized as a simple, flexible and net-shape capable method in high-energy ball milling (HEBM), can successfully employ the ball-powder-ball collisions to disperse reinforcement uniformly^4,24^. However, the PM process cannot be considered as a perfect process for a uniform dispersion of nanoparticles within matrix when there is a visible difference between the size of reinforcement and the matrix^29,30^. In other words, nanoparticles tend to agglomerate during solid-state process such as PM^31^, obstructing the formability of the composites in the subsequent processes^32–34^. Thus, it is crucial to design a simple and effective method to uniformly disperse nanoparticles within an aluminum metal matrix through which adequate deformation of the bulk composites in the subsequent forming process can be achieved, enhancing mechanical properties^35^.

In recent years, flake powder metallurgy (FPM) has emerged as a sophisticated route to fabricate MMNCs. Morsi and Esawi^36^ showed the flattening of aluminum particles at the beginning of mechanical milling, forming a flake shape. Additionally, Hesabi *et al*.^37^ confirmed the transformation of spherical particles into a flake shape, indicating the advantages of the flake shape over the spherical shape to accommodate nanoparticles of hard reinforcement. Furthermore, Tjong^19^ observed that increasing the milling time, for example, by 24 h, leads to the fragmentation of the formed flaky particles. In 2011, for the first time, Jiang *et al*.^38^ reported a new approach they called it flake powder metallurgy, in which aluminum flakes with formed Al_2_O_3_ skins on top can be used as bases to compose new material in a definitive manner, rather than leaving it to develop arbitrarily as in the case of spherical shape powder. They also defined the three steps of a typical FPM route: preparation of flake powder, production of composite powder, and consolidation of bulk composite. In^39^, they extended their previous work to produce strong and ductile Al/CNT nanocomposite, achieving a strength of 435 MPa and ductility of 0.06. These very superior results were attributed to the ability of their approach to solve the problem encountered with conventional PM. Moreover, Kai *et al*.^40^ employed FPM to produce a strong and ductile Al/B_4_C composite, achieving a strength of 364 MPa and ductility of approximately 0.09, which were ascribed to the effect of an enriched normalized strain hardening rate for enhanced ductility. In^35^, FPM was utilized, with Al/B_4_C confirming its applicability in uniform dispersal of a high volume fraction of reinforcements, achieving high strength without compromising the ductility. Furthermore, in a review paper, Fan *et al*.^41^ assured that a strong and ductile composite can be fabricated by a perfectly designed FPM. They also pointed out some issues regarding the maturity of FPM that are yet to be investigated. In fact, a number of studies^42–49^ have been conducted to explore different aspects of FPM in recent years. For example, Varol *et al*.^43^ succeeded in combining the first two steps of FPM into one step.

In the advancement of flake powder metallurgy, in 2017, Xu *et al*.^29^ suggested and implemented a task allocation strategy to enhance ductility while maintaining high strength. They called this route shift-speed ball milling (SSBM), in which a combination of low-speed and high-speed was utilized in the same process to obtain the advantageous of both speeds. They claimed that their findings were very encouraging and attributed their results to achieving good dispersion and interfacial bonding.

Consequently, the implementation of a shift-speed process in FPM was studied with different composites, with primarily CNT and graphene as reinforcements in^49–51^. However, it is necessary to point out that the shift-speed in the previous studies involved using only two different speeds. Thus, the effect of a variety of speeds is a compelling consideration to explore. From this point of view, this study examined the effect of implementing different fabrication routes by shifting the ball milling speed up and down three times. Furthermore, for the first time, SiC was applied to reinforce the aluminum matrix in shift-speed ball milling.

## Materials and Methods

### Fabrication routes

Three different processing conditions SSBMr, SSBMd, and SSBMu with carefully designed ball-milling processes were utilized to examine the effectiveness of shifting the ball milling speed in achieving uniform distribution and homogenous dispersion of nanoparticle reinforcement in aluminum MMCs. In addition, the mechanisms of these different fabrication routes, described in Table 1, were explored. Carreño-Gallardo *et al*.^52^ pointed out the best hardness and strength were achieved at 2.5 wt.%. Thus, in the fabrication process, 2 wt.% of nanosized β-SiC with 95% purity and average size of 60 nm, purchased from Alfa Aesar (Germany), were mixed with aluminum fine powder of 98% purity and average particle size of 30 µm, purchased from Loba Chemie (India). The ball-to-powder ratio was set to 15:1. The powder was ball milled continuously, alternating between 15 min of milling and 15 min of stoppage to allow for cooling. To avoid severe cold-welding, 2 wt.% of stearic acid was included in the mixture during ball milling. Using steric acid with air atmosphere has been proved to be more favorable in reducing cold welding^53^.The ball milling process was accomplished in air atmosphere using a planetary ball mill (Pulverisette 7, Fritsch, Idar-Oberstein, Germany) with milling jars of 80 ml in size. Five grams of powder mixture was used per batch.

**Table 1:** Designation system.

Route name	Ball milling process stages					
	Stage1		Stage2		Stage 3	
	Speed (rpm)	Time (h)	Speed (rpm)	Time (h)	Speed (rpm)	Time (h)
SSBMr	150	8	300	4	n/a	n/a
SSBMd	150	8	300	4	150	2
SSBMu	150	8	300	4	450	1

First, the reference route was SSBMr, containing two speeds. The subscript “r” refers to regular. In this route, the initial speed in the ball milling process was 150 rpm, which was utilized for 8 h of actual milling time; then, the speed was shifted to 300 rpm for 4 h of actual milling time. Next, in the SSBMd route, the process was modified to include 3 speeds, adding to the SSBMr process a downshift in speed to 150 rpm for 2 h to examine the effect of downshifting the speed. The subscript “d” refers to the downshifting. Finally, in the SSBMu, this route is similar to the second route with the last step modified to represent an upshift in speed up to 450 rpm for 1 h of actual milling time to examine the effect of upshifting the speed. The subscript “u” refers to upshifting. The last two routes have never been applied before and are the original ideas of this research.

The ball milled powder was consolidated by a high-frequency induction heat sintering furnace (HFIHS) from ELTek Co., South Korea. In this process, the compaction and sintering processes are conducted simultaneously over a short period of time. In HFIHS, the temperature is generally held approximately 20% lower than the solidus temperature of the base materials being processed, which maintains the metal elements in their solid state and avoids the formation of a liquid phase.

**Figure 1:**
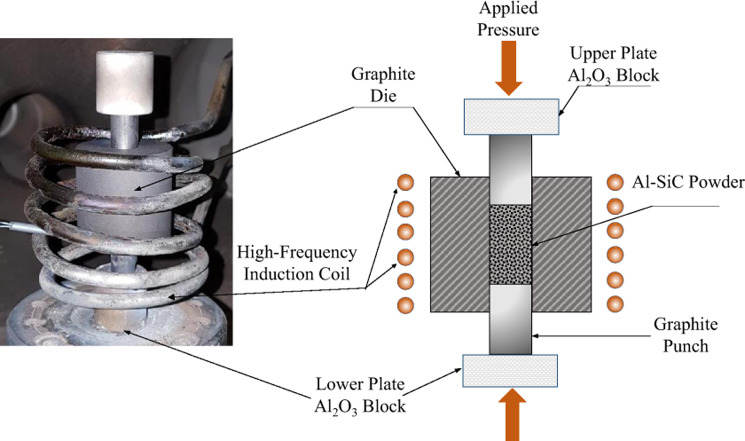
Schematic of high frequency induction heat sintering furnace.

HFIHS uses a 10-mm inner diameter graphite die, indicated in Fig. 1, to consolidate the powder sample. The ball milled powder is loaded into the graphite die in the evacuated chamber and a uniaxial pressure is applied through the sintering process. The required heat for the sintering process is generated by applying a strong magnetic field in the electrically conducting die and the sample itself. A pyrometer focused on the die surface is used to measure the temperatures. The HFIHS parameters used in this study are shown in Table 2. These consolidation parameters were fixed for all process in this study.

**Table 2:** Sintering parameters.

Parameter	Used value	Unit
Vacuum level	1 × 10^−3^	Torr
Heating rate	150	°C/min
Applied pressure	40	MPa
Duration time	5	min
Applied temperature	570	°C

### Investigation method

Several investigations were conducted throughout the fabrication process to yield insights into the SSBM routes, which will serve as a theoretical basis. The raw materials were characterized using FESEM and HRTEM. From these investigations, the characteristics of the powder were described, and the size of particles were measured. After ball milling, FESEM and EDS were employed to determine the extent to which the morphology of the powder had evolved, shape and sizes of particles had changed, and homogeneity of distribution of the reinforcement had been achieved. Furthermore, X-ray diffractometer D-8 Discover, Bruker, Germany, was used to investigate the powder and bulk composite. The system has a rotating anode source with a copper target and a wide-angle powder goniometer. The source was operated at 40 kV using filtered Cu Kα radiation (λ = 1.5406 Å) and 40 mA. Rietveld refinement was applied on the obtained scans. Scherrer formula was used for calculation of crystallite size. After consolidation, the bulk composites were polished, and their sizes and actual densities were measured. The actual densities were measured using Archimedes’ method. After consolidation, the microhardness of the samples was measured. The hardness of the polished bulk composites was measured using the Vickers hardness tester (WOLPERT UH930, Wilson Hardness, Shanghai, China) at a 5-kN load. The compression test was performed on Instron 5582 Microtester according to ASTM: E9-89a at room temperature with a strain rate of 10^−3^/s. The test specimens were of 10 mm diameter with aspect ratio of (L/d ∼ 1:1.5). In order to avoid the barreling effect, a grease lubrication was used. The obtained stress-strain graphs were recorded using Bluehill2 software.

### Illustration of the fabrication route mechanism

**Figure 2:**
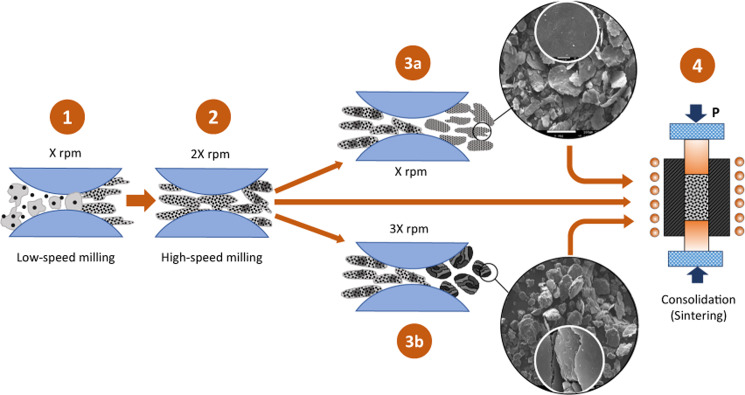
Illustrated mechanisms of fabrication routes.

The overall strategy implemented in this research can be divided into four stages, schematically shown in Fig. 2. As shown in Fig. 2, all routes started at stage 1, the low-speed ball milling stage. This stage was studied in previous investigations and has been proven to produce flaky shape particles, allowing reinforcements to be distributed uniformly in the matrix powder^41^.This is, in fact, the basis for the flake powder metallurgy route in the literature. The behind-the-scenes mechanism of this stage involves the flattening of metal matrix particles due to continuous ball-powder-ball collisions, resulting in an increase in the compatibility of the matrix particles to accommodate the reinforcements nanoparticles by transforming the shapes of the particles of the base matrix from irregular spherical to flake-like. In addition, this stage includes the fragmentation of agglomerated reinforcements and their subsequent uniform distribution between the matrix particles, readying them for the next stage.

Next, all routes pass through stage 2, which can be called the high-speed ball milling stage. In this stage, the ball-powder-ball collisions are increased, inducing high compressive force, which causes the hard SiC nanoparticles to penetrate the ductile Al flakes. Moreover, the edges of the Al flakes are broken and laminated onto the surface of the flakes. It should be noted that stages 1 and 2 together constitute the strategy called shift-speed ball milling, as mentioned before. By the end of this stage, the powder of the SSBMr route is ready to proceed directly to stage 4, skipping stage 3.

Stage 3 is the novelty of this study and contains two paths, 3a and 3b. The route SSBMd differs from SSBMr by passing through path 3a. In this path, the ball milling continues with the downshift in speed to the low speed again but for a shorter time. This downshifting in speed results in the smoothing of the surface and reduction in the thickness of the flakes. This behavior was ascribed to the reduction in the compressive force while the shearing force is maintained. This leads to a number of noted effects. For example, laminated small particles and penetrating SiC nanoparticles that are not strongly bonded become detached from the large flakes. In contrast, those that became more than halfway bonded, completed their way to full attachment. Additionally, the micro-rolling of the balls causes the flake thickness to decrease. The resulting powder at the end of this path is the SSBMd powder, which proceeds to stage 4 to complete the process route.

The other novel technique in this study is the SSBMu route. This route differs from SSBMr by passing through path 3b. In this path, the speed of ball milling is upshifted by up to 3 times the low speed for a very short time. The ball milling intensity increases, inducing a higher compressive force and resulting in more accelerated fracturing and welding. Consequently, the morphology of this powder is converted from a flake shape to an equiaxed shape. This confirms that upshifting the speed allowed the ball milling process to reach a steady state between fracturing and welding. The powder of the SSBMu route is ready to be consolidated in stage 4.

Stage 4 involves the consolidation of the produced powder using HFIHS. This technique is quick, simple, and capable of controlling grain growth effectively^54,55^.

## Results and Discussion

### Raw powder characterization

**Figure 3:**
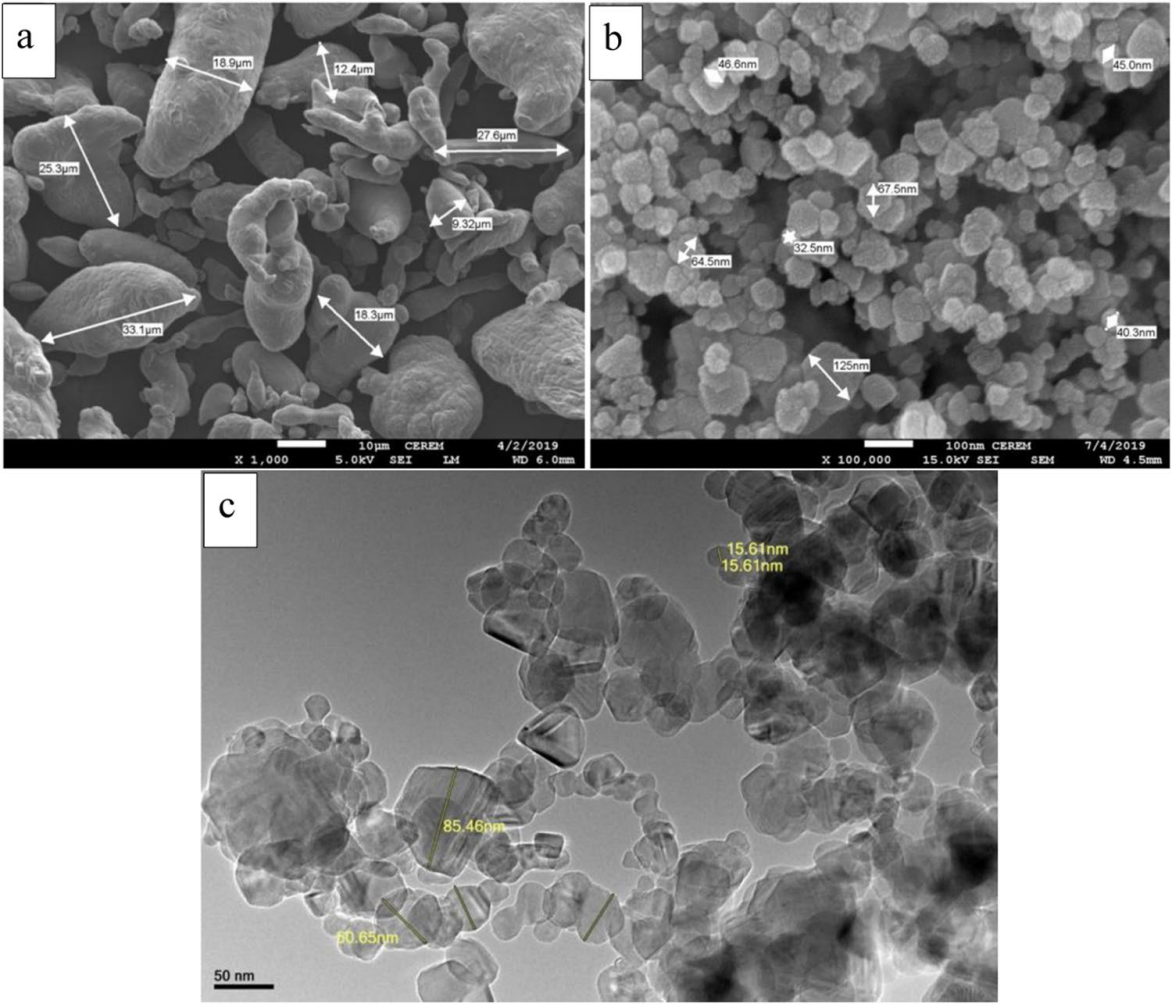
FESEM images of (**a**) Al powders and (**b**) SiC powder, and HRTEM image of (**c**) SiC powder.

The FESEM and HRTEM images in Fig. 3 show the characteristics of the as-received powder of both Al and SiC. Some measured particles of both powders are presented in Fig. 3 confirming the measurement provided by the supplier. Additionally, the agglomeration of SiC particles is clearly seen in Fig. 3(b).

### Ball milled powder characterization

**Figure 4:**
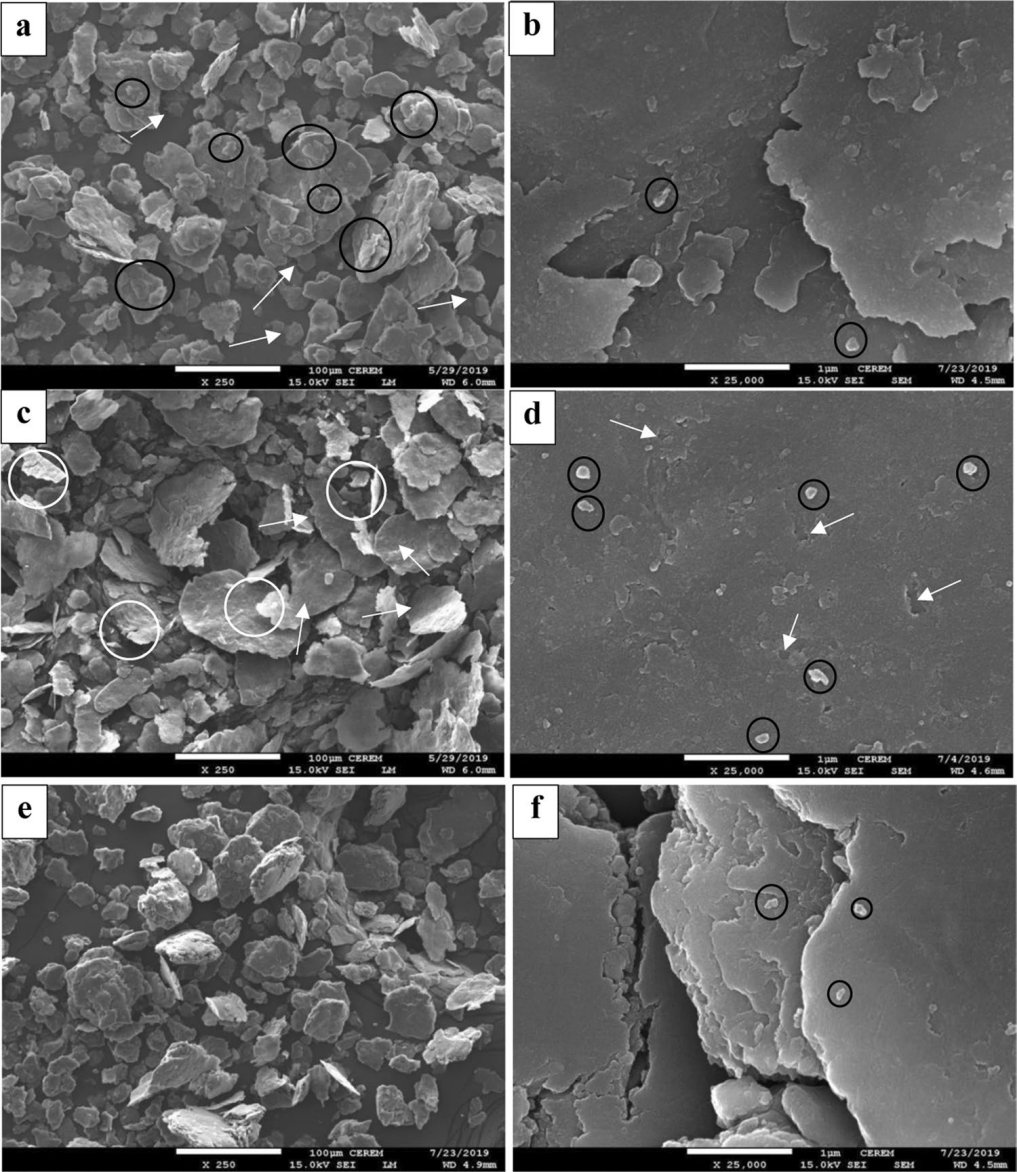
FESEM images for powder produced by (**a**,**b**) SSBMr, (**c**,**d**) SSBMd and (**e**,**f**) SSBMu.

One of the keys to understanding the ball milling process is the morphological analysis of the powder, which can reveal the change in the powders morphology as result of changing the milling process parameters. The morphological analysis can help investigate the manner in which the reinforcement is distributed, and the size of the particles. All these investigations play a very important role in understanding how to achieve enhanced mechanical and physical properties in the produced composite. From this point of view, FESEM was used to obtain images of the ball milled powder representing the three designed routes in this study. Figure 4 shows the morphology of the ball milled powder.

Figure 4(a) shows the characteristics of the powder produced by the SSBMr route. The main characteristic of this powder is the uniform size and flake shape of the majority of the powder particles. Additionally, there is a small amount of broken small particles, which are indicated by the white arrows in Fig. 4(a). Moreover, laminations of soft broken Al particles are depicted on top of some of the large Al flakes, which can be seen inside the black circles in Fig. 4(a). The agglomeration of SiC in the powder produced by this route is very low, and only a few particles exit in each cluster, as indicated by the black circles in Fig. 4(b).

In contrast, in the SSBMd route, the downshifting of the milling speed minimized the compressive force while maintaining the shearing force, resulting in the removal of the laminated particles that were still not strongly bonded and the smoothing of the surfaces, as clearly seen in Fig. 4(c). Additional evidence supporting this suggestion is found in Fig. 4(d), in which the white arrows pointed toward the voids are suggested to represent areas previously occupied by SiC nanoparticles that penetrated the Al flakes, and its removal was due to the shearing force as result of shifting the ball milling speed downward. Moreover, there are some voids that started to heal, proving the idea of surface smoothing.

Finally, in the SSBMu route, the morphology of the powder is completely different, as clearly seen in Fig. 4(e), where the transformation of the flake shapes into equiaxed shapes is evident. This confirms that the powder has reached a steady state between fracturing and welding^56^. The size of the particles becomes larger owing to the welding of some Al flakes together. This is a result of the increased compressive force due to upshifting the ball milling speed. Additionally, the reinforcement agglomeration is very low and, again, there are only few particles in each cluster, as seen Fig. 4(f). Furthermore, the number of clusters is very small in comparison with those for SSBMd. It is worthy to mention here that the low number of SiC nanoparticles in the clusters indicates a homogenous distribution of SiC nanoparticles in the produced powder by the three routes implemented in this study.

**Figure 5:**
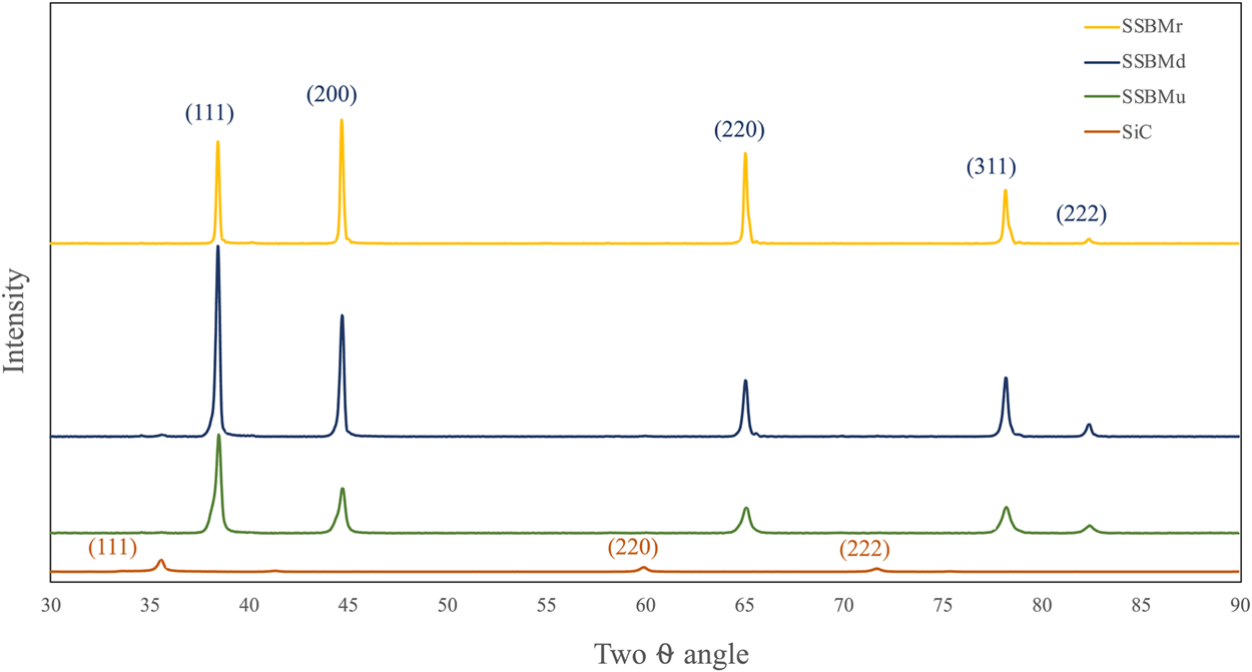
XRD patterns for the ball milled powder.

XRD analysis of the powder can be used to investigate the presence of SiC in the ball milled powder. Thus, the XRD patterns of the ball milled powder by the three routes are presented in Fig. 5. First, the SiC spectrum is presented for reference. In this spectrum, the three main peaks of SiC are clearly seen. In the ball milled powder patterns, the Al peaks are broad, and their intensities are diminished.

In the sample produced by SSBMr, the broadening is lower than that in the other samples, indicating that the crystallite size of this sample is larger than others. Moreover, in the sample produced via SSBMd, the diminishing in Al peak intensity is lower than that in the other samples, indicating that the dislocation density in this sample is high in comparison with the other samples. Additionally, in this sample, the SiC peak is clearer, confirming the agglomeration that was mentioned before.

**Figure 6:**
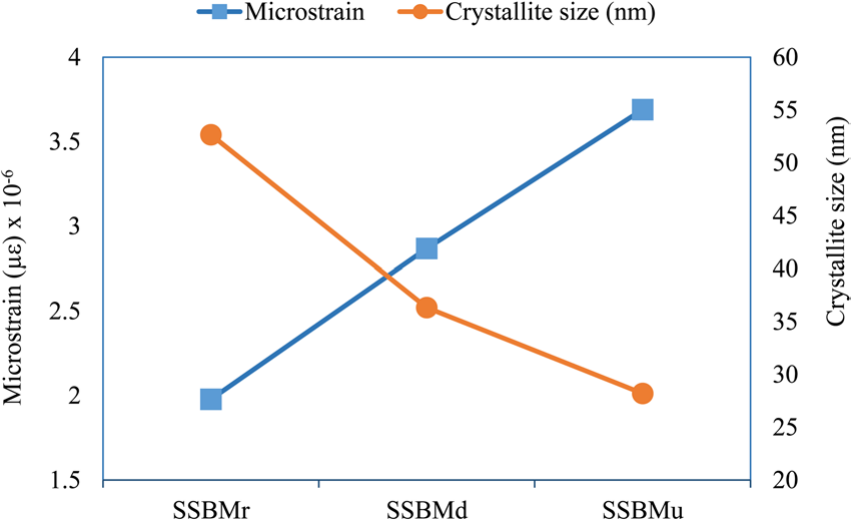
Crystallite size and microstrain for the ball milled powder.

In contrast to the samples produced by SSBMr and SSBMd, the pattern of the sample produced via SSBMu shows the most diminished peak and the highest broadening, confirming the smallest crystallite size and the highest microstrain as shown in Fig. 6. This assures the effectiveness of this route in restricting grain growth and preventing dislocation movement, resulting in better mechanical properties as will be presented later. Finally, the investigation of the XRD patterns shows that there are no extra peaks, which suggest that no other phases are formed^13^.

The crystallite size and microstrain calculations were based on the peak angle $2{\boldsymbol{\theta }}$ and full width at have maximum (FWHM) obtained directly from DIFFRAC.EVALUATION PACKAGE software. Using these values, the crystallite size (D) was calculated as following:

$${\rm{D}}=\frac{{\rm{k}}{\rm{\lambda }}}{{\rm{\beta }}\,\cos \,{\rm{\theta }}}\,{\rm{where}}\,{\rm{\beta }}={\rm{F}}{\rm{W}}{\rm{H}}{\rm{M}},\,{\rm{k}}=0.89,\,{\rm{and}}\,{\rm{\lambda }}=0.15406$$

Additionally, the microstrain (${\boldsymbol{\varepsilon }}$) was calculated as following:

$${\boldsymbol{\varepsilon }}=\frac{{\boldsymbol{\beta }}}{4\,\tan \,{\rm{\theta }}}$$

To ensure homogenous distribution of SiC nanoparticles in the produced powder by the three routes implemented in this study, an elemental mapping was generated using EDS (aluminum in grey, carbon in red, and silicon in yellow). In elemental mapping, SiC is represented by its contents Si (silicon) and C (carbon).

**Figure 7:**
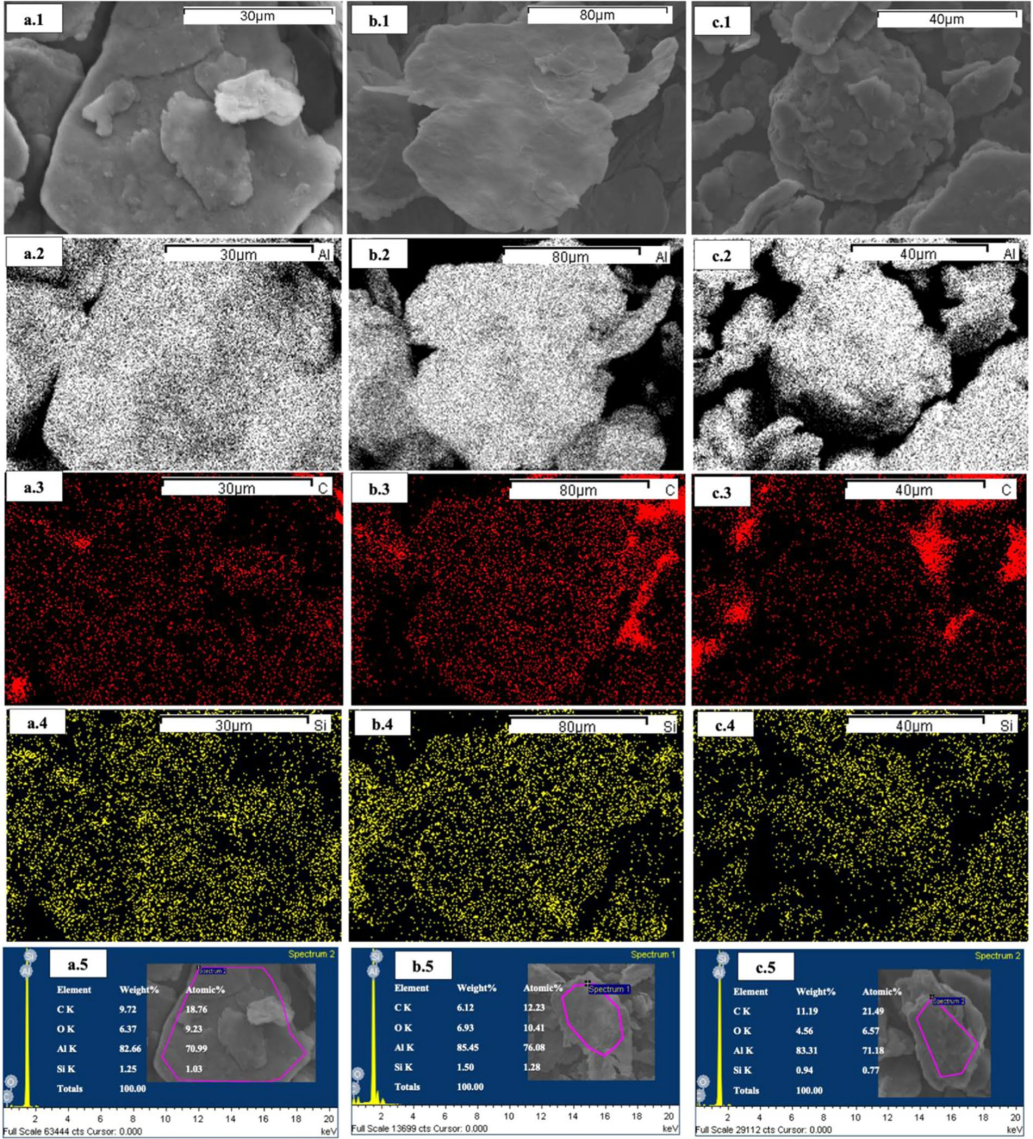
EDS elemental mapping for (**a**) SSBMr, (**b**) SSBMd and (**c**) SSBMu.

Figure 7(a–c) show that the SiC has a homogenous distribution in all the three routes. This is actually expected because all the three routes rely on the effectiveness of the preparation of Al flakes at the first stage to accommodate the SiC nanoparticles. The SiC nanoparticles get dispersed between Al flaky particles owing to the low speed at the beginning. The reset of the process in all routes has no adverse effect on this homogenous distribution because the three routes have been designed carefully to maintain the advantages of flake powder metallurgy.

### Physical properties of the products

**Figure 8:**
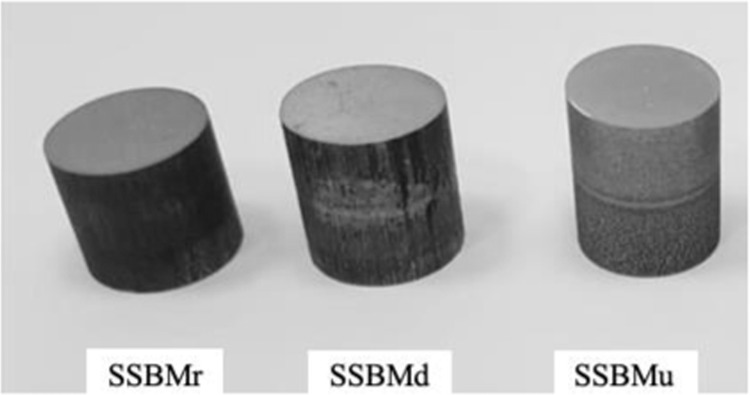
Set of consolidated bulk composites.

The ball milled powder was consolidated using HFIHS system. A set of the produced samples are presented in Fig. 8. The samples are identified by the name of the route used to produce them. The samples have a 10-mm diameter, and their other physical properties are presented in Table 3.

**Table 3:** Physical properties of the produ cts.

Route name	Density			
	Calculated (g/cm^3^)	Archimedes (g/cm^3^)	Theoretical (g/cm^3^)	Relative (average) (%)
SSBMr	2.502	2.513	2.71	93.1
SSBMd	2.612	2.631	2.71	97.1
SSBMu	2.626	2.651	2.71	97.4

The low relative density of the sample produced by SSBMr can be attributed to the larger particle size with flake shape as result of lower ball milling time. The flake shape has lower packing ability and the lower ball milling time results in lower densification rate as stated in^56^. This means that this sample has some porosity in comparison with the other samples. However, the highest relative density in the sample produced via SSBMu confirms the effectiveness of the SSBMu route on producing a suitable shape of particles for consolidation process.

**Figure 9:**
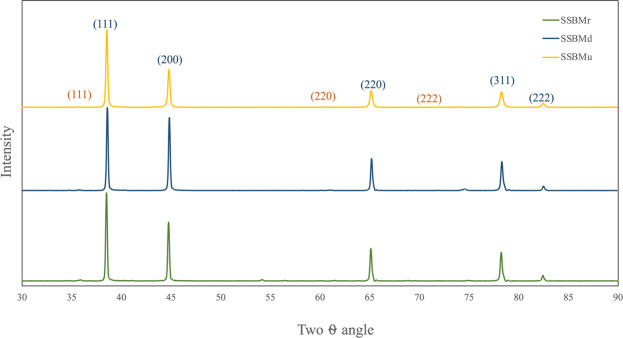
XRD pattern for Al/SiC bulk composites.

XRD was employed again to investigate the produced bulk composites. The patterns of the three samples are presented in Fig. 9.

**Figure 10:**
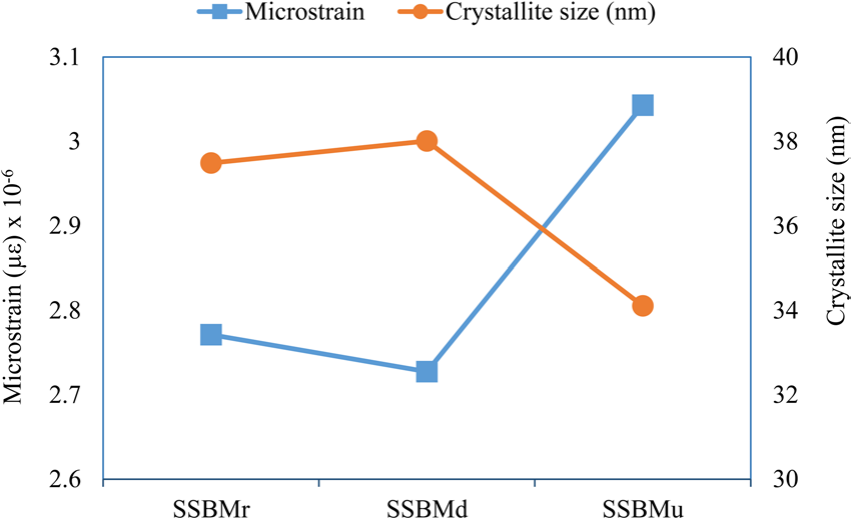
Crystallite size and microstrain for bulk composites.

From these XRD patterns, SiC peaks can be identified, confirming its homogenous distribution in all samples. Moreover, there is a small peak near 54° and 56° in the pattern of the sample produced via SSBMr route, suggesting a formation of new phases during the consolidation process. These phases could be Al_4_C_3_ and/or Si. The reason behind this is the lack of sufficient bonding in this route, as mentioned before. However, these phases are not seen in the other samples. The crystallite size and microstrain of bulk composites are presented in Fig. 10.

The crystallite size of the bulk composites shows interesting results where is there a refining in the grain of the sample produced by SSBMr in comparison with its value when it was in ball milled powder form in Fig. 5. This refinement is not seen in the other samples as their crystallite size in the powder form is already low. This is attributed to the advanced sintering process, which is in agreement with the results obtained in by Liu *et al*.^57^, who stated that the advanced sintering resulted in grain refinement if the starting powder have larger grain size but this is not applicable when the grain size is already low.

## Mechanical Properties

### Hardness results

**Figure 11:**
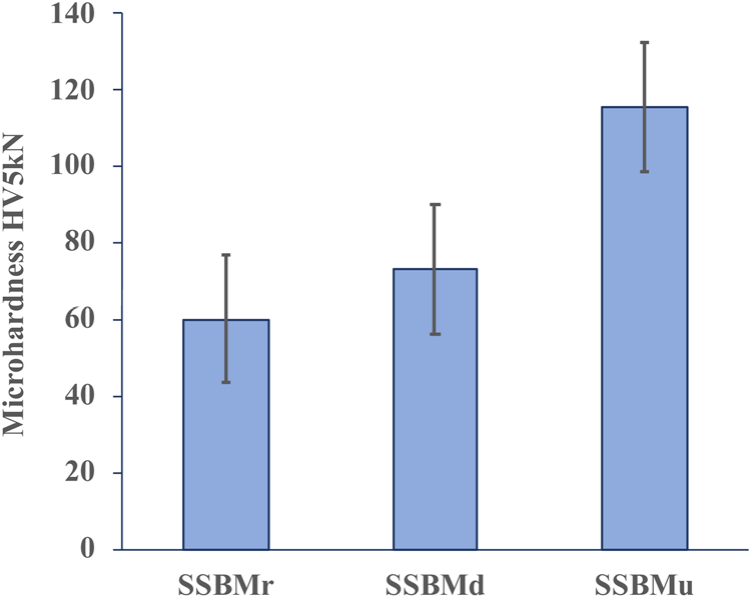
Hardness results.

The average microhardness values of 60.33, 73.23, and 115.60 HV5kN were obtained for Al reinforced with 2 wt.% SiC, and correspond to SSBMr, SSBMd, and SSBMu respectively. The microhardness results are presented in Fig. 11.

The implemented routes clearly have different effects on the obtained microhardness results. The lower hardness of the SSBMr sample can be attributed to the lower ball milling time in this route compared with the other two routes. In contrast, the composite produced by SSBMd has a slightly enhanced hardness due to further ball milling, even though this further ball milling was at low speed and for a shorter time. Moreover, huge enhancement can be seen in the bulk composite produced by SSBMu, which is 57% and 92% higher than that produced by SSBMd and SSBMr, respectively. This can be attributed to the unprecedented design of the SSBMu route, taking the advantages of flake powder metallurgy via the shift in speed and heading toward the steady state. This new advancement in ball milling combines the high interfacial bonding of the Al/SiC, the uniform distribution of SiC, and the task allocation for SiC nanoparticles to block the motion of dislocations.

### Compression results

**Figure 12:**
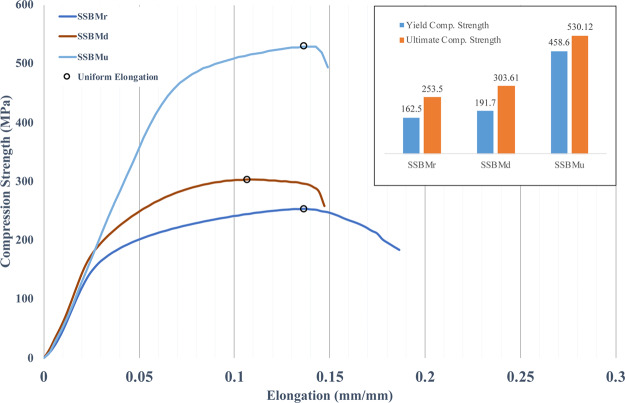
stress-strain uniaxial compression curves.

The stress-strain uniaxial compression curves of 2 wt.% SiC/Al composites are prepared by different routes are shown in Fig. 12.

The analysis of the curves in Fig. 12 has shown that SSBMr has the highest total elongation. However, for measuring ductility in nanostructured materials, uniform elongation is more appropriate than total elongation because the latter is more affected by gauge length than the former^58^.The uniform elongations in Fig. 12 are marked by black circles for each route. SSBMr is the regular shift speed, but it is used in this study for first time to fabricate Al/SiC and, for first time, with HFIHS. Thus, its results will be used as a baseline to determine the enhancement in mechanical properties by the new original routes developed in this study. Thus, the SSBMd route as shown in Fig. 12 has resulted in enhanced strength but at the cost of ductility. Total elongation and uniform elongation have been reduced in this route. This is can be attributed to the increase of brittleness in the Al particles due to the prolonged milling time but without sufficient milling speed to induce more work hardening, which is favored for uniform elongation.

The excellent design of SSBMu has resulted in enhancing the strength to 520 MPa, as seen in the SSBMu curve, which is approximately 101% higher than that for SSBMr with 0% loss in uniform elongation. This unprecedented result can be attributed to the steps included in this route. These steps start with lower speed milling, resulting in the control of the preparation of the flake shape in the base matrix particles and uniform distribution of the reinforcement particles. Then, high-speed milling follows to take advantage of the higher compressive force to laminate some of the broken Al particles on top of the large particles. Following this, an even higher speed was employed to strengthen the bonding between the base matrix particles and reinforcement particles and reach the steady state for ball milling.

Yield compressive strength and ultimate compressive strength are presented as inset in Fig. 12. The bar chart shows major enhancement in the strength properties for the SSBMu sample compared with SSBMr and SSBMd. This inset shows the yield and ultimate strengths of SSBMu as180% and 101% respectively higher than those for SSBMr.

## Conclusions

Tuned shift-speed ball milling routes were proposed to produce Al/SiC composites with high strength and good ductility. The suggested mechanisms for each route were illustrated based on the scientific background of powder metallurgy. The analysis of the morphological evolutions of the resulting powders has fairly confirmed the explained mechanisms. This uses the shift-speed ball milling method demonstrated before to be effective in addition to two new routes as modifications of this route. The conclusions can be summarized as follows:

- The regular shift-speed ball milling SSBMr was the basis for comparison. In this route, the underlying mechanism of particle deformation and reinforcement dispersion involves the conversion of Al particles into flake shapes, with some fractured particles laminated on top of them and a uniform dispersion of reinforcement, but lacking sufficient bonding. The mechanical properties of the composites produced by this route were taken as bases with which other samples to be compared.
- In the second route (SSBMd), the deformation and dispersion mechanism were suggested. These were confirmed by morphological analysis to be the removal of some laminated particles and smoothing of the surface as result of decreasing the compressive force. The mechanical strength of the composites produced by this route has shown slight improvement but at the cost of uniform elongation. This is ascribed to the high brittleness due to the extension of ball milling time with inadequate work hardening.
- In the third route (SSBMu), the proposed mechanism of the particle deformation and reinforcement dispersion was confirmed. The morphological analysis of the ball milled powder from this route has shown that the powder transforms into an equiaxed shape, confirming that the ball milling process has reached a steady state. The distribution of the nanoreinforcement is highly homogeneous, and the bonding is very strong. This is also confirmed by the high enhancement in the yield compressive strength (180%), ultimate compressive strength (101%), and hardness (92%), with 0% loss in uniform elongation compared with SSBMr.

These findings confirm that FPM routes, when carefully designed, could provide a breakthrough in the synthesis of the required materials.
